# Assessing the causal association between 25‐hydroxyvitamin D and the risk of oral and oropharyngeal cancer using Mendelian randomization

**DOI:** 10.1002/ijc.31377

**Published:** 2018-07-30

**Authors:** Tom Dudding, Mattias Johansson, Steven J. Thomas, Paul Brennan, Richard M. Martin, Nicholas J. Timpson

**Affiliations:** ^1^ MRC Integrative Epidemiology Unit University of Bristol Bristol United Kingdom; ^2^ Bristol Medical School, Population Health Sciences University of Bristol Bristol United Kingdom; ^3^ Bristol Dental School University of Bristol Bristol United Kingdom; ^4^ International Agency for Research on Cancer Section of Genetics–Genetic Epidemiology Group Lyon France; ^5^ National Institute for Health Research (NIHR) Bristol Biomedical Research Centre University Hospitals Bristol NHS Foundation Trust and the University of Bristol Bristol United Kingdom

**Keywords:** oral cancer, oropharyngeal cancer, 25‐hydroxyvitamin D, Mendelian randomization

## Abstract

Circulating 25‐hydroxyvitamin D (25OHD) is an appealing potential intervention for cancer risk and has been associated with oral and oropharyngeal cancer risk but evidence is inconsistent. The availability of genetic variants, uncorrelated with known confounders, but predictive of 25OHD and genetic data in a large oral and oropharyngeal cancer collaboration aids causal inference when assessing this association. A total of 5,133 oral and oropharyngeal cancer cases and 5,984 controls with genetic data were included in the study. Participants were based in Europe, North America and South America and were part of the Genetic Associations and Mechanisms in Oncology (GAME‐ON) Network. Five genetic variants reliably associated with circulating 25OHD were used to create a relative genetic measure of 25OHD. In the absence of measured 25OHD, two‐sample Mendelian randomization using individual level outcome data were used to estimate causal odds ratios (OR) for cancer case status per standard deviation increase in log25OHD. Analyses were replicated in an independent population‐based cohort (UK Biobank). In the GAME‐ON study, there was little evidence of a causal association between circulating 25OHD and oral cancer (OR = 0.86 [0.68;1.09], *p* = 0.22), oropharyngeal cancer (OR = 1.28 [0.72;2.26], *p* = 0.40) or when sites were combined (OR = 1.01 [0.74;1.40], *p* = 0.93). Replication in UK Biobank and pooled estimates produced similar results. Our study suggests that a clinically relevant protective effect of 25OHD on oral and oropharyngeal cancer risk is unlikely and supplementation of the general population with 25OHD is unlikely to be beneficial in preventing these cancers.

## Abstract 2

**What's new?**

Evidence for a role of vitamin D in head and neck cancers is contradictory, and randomized controlled trials are not well suited to investigate the potential effect of 25‐hydroxyvitamin D (25OHD) supplementation. For the first time, this study used Mendelian randomization with genetic variants associated with 25OHD and uncorrelated with known confounders to derive the causal effect of 25OHD on oral cavity and oropharyngeal cancer risk. The findings suggest that a clinically‐relevant protective effect of 25OHD on oral and oropharyngeal cancer risk is unlikely, making supplementation of the general population with 25OHD unlikely to be beneficial in preventing these cancers.

**Abbreviations**
1,25OH_2_D: 1,25 di‐hydroxyvitamin D
25OHD: 25‐hydroxyvitamin D
CI: confidence interval
CEU ancestry: northern Europeans from Utah
GAME‐ON: Genetic Associations and Mechanisms in Oncology
GWAS: Genome Wide Association Study
HNC: head and neck cancer
MAF: minor allele frequency
MR: Mendelian randomization
OR: odds ratio
QC: quality control
RCT: randomized controlled trial
SNP: single nucleotide polymorphism

## Introduction

Each year, there are approximately 300,000 new oral cavity and 230,000 new oropharyngeal cancers worldwide.^1^ Squamous cell cancers of the head and neck (HNC) are heterogeneous and oral and oropharyngeal cancers have differing aetiology. Alcohol and tobacco use are the major risk factors for oral and oropharyngeal cancers,^2^, ^3^ explaining approximately 65–70% of the population attributable risk.^4^ Infection with human papillomavirus (HPV) has emerged as an important risk factor, particularly in oropharyngeal cancers.^5^ The disease burden of these cancers remains high despite reductions in the prevalence of tobacco and alcohol use. Oropharyngeal cancer incidence continues to increase in the UK despite no increase in HPV infection in the past 10 years;^6^ therefore, identification of other modifiable risk factors remains important.

Vitamin D is predominantly synthesised by exposure to ultra‐violet light. The inactive metabolite (25‐hydroxyvitamin D [25OHD]) is the most commonly clinically measured and supplemented form and is correlated with the active form, 1,25‐dihydroxyvitamin D (1,25OH_2_D). An endocrine feedback mechanism controls activation of 25OHD to 1,25OH_2_D in the kidneys which is important for calcium and phosphate balance.^7^ Evidence from mechanistic studies suggests a protective role of 1,25OH_2_D on cancer incidence and progression,^8^, ^9^ and this may act by the same endocrine mechanism or via paracrine effects at the cancer site. It is not clear whether higher circulating 25OHD is itself protective against cancer or whether other factors such as the ratio of 25OHD to 1,25OH_2_D at the cancer site add further complexity (Tan V et al., Submitted for publication).

Evidence for a role of vitamin D in HNCs is contradictory. In an observational study of tobacco‐related tumours, participants with 50% lower circulating 25OHD were more likely to develop HNC after accounting for available potential confounders (Hazard ratio = 1.44 [95% confidence interval (CI): 1.19, 1.73]).^10^ A recent prospective European cohort study also found strong evidence for an inverse association between circulating 25OHD on HNC risk (odds ratio [OR] per doubling of circulating 25OHD concentration = 0.69 [95% CI: 0.56, 0.87], *p* = 9×10^−4^).^11^ However, Arem *et al*.^12^ and Skaaby *et al*.^13^ found no convincing evidence of a protective effect of higher 25OHD levels on HNC.

Well‐designed randomized studies of 25OHD supplementation are required to identify whether there is a true causal link with cancer incidence. However, randomized controlled trials (RCTs) are not well suited to investigate the effect of 25OHD supplementation on rare diseases. Long follow‐up duration, non‐adherence to intervention or control regime and an unknown optimal supplementation dose limit such trials^14^ meaning they are prohibitively expensive and lack power to detect relatively small but potentially clinically important effects.

In the context of this lack of evidence in the literature, Mendelian randomization (MR) can help assess whether there is a causal association between 25OHD and oral and oropharyngeal cancer risk by utilising genetic data, even in studies where 25OHD itself has not been measured. MR uses genetic variants known to be reliably associated with a risk factor of interest (*e.g.,* 25OHD) to derive estimates of the causal effect of that risk factor on health outcomes (*e.g.,* oral cavity and oropharyngeal cancer risk).^15^, ^16^, ^17^ Other studies have used these methods to provide evidence for causal associations of 25OHD with health outcomes including other cancers^18^ and multiple sclerosis.^19^

Our study aims to assess the causal association between vitamin D and oral and oropharyngeal cancer risk and estimate the size of any effect using MR.

## Methods

### Participants and genotyping

#### GAME‐ON

The study comprised 6,034 HNC cases and 6,585 controls from studies which were part of the Genetic Associations and Mechanisms in Oncology (GAME‐ON) Network. Cases and controls from 10 different case–control studies were included, as well as from the European cohort study (EPIC) and cases from a UK case series (Head and Neck 5000 [HN5000]). Details of the studies included have been described previously.^20^ Informed consent was obtained for all participants and studies were approved by respective institutional review boards. For our study, cancer cases of interest comprised the following ICD codes: oral cavity (C02.0–C02.9, C03.0–C03.9, C04.0–C04.9 and C05.0–C06.9) and oropharynx (C01.9, C02.4 and C09.0–C10.9). DNA extraction, genotyping, quality control and imputation has been described previously.^20^ The study population included participants from Europe, North America and South America. To reduce the effect of heterogeneity across these regions, only participants with >70% CEU ancestry were included in analyses (*n* = 11,117). These consisted of 5,133 cases (oral *n* = 2,700 and oropharyngeal *n* = 2,433) and 5,984 controls (Table 1).

**Table 1:** GAME‐ON participant summaries

	All, *n* (%)	Case, *n* (%)	Control, *n* (%)
	*N* = 11,117	*N* = 5,133	*N* = 5,984
Site			
Oral		2,700 (52.6)	
Oropharyngeal		2,433 (47.4)	
Age (years)			
≤50	2,217 (19.9)	1,071 (20.9)	1,146 (19.2)
50–<60	3,443 (31.0)	1,696 (33.0)	1,747 (29.2)
60–<70	3,348 (30.1)	1,504 (29.3)	1,844 (30.8)
≥70	2,108 (19.0)	861 (16.8)	1,247 (20.8)
Missing	1 (0.01)	1 (0.02)	0 (0.00)
Sex			
Male	7,680 (69.1)	3,798 (74.0)	3,882 (64.9)
Female	3,437 (30.9)	1,335 (26.0)	2,102 (35.1)
Smoking status			
Never	3,302 (29.7)	1,002 (19.5)	2,300 (38.4)
Previous	3,655 (32.9)	1,590 (31.0)	2,065 (34.5)
Current	3,300 (29.7)	2,019 (39.3)	1,281 (21.4)
Missing	860 (7.74)	522 (10.2)	338 (5.65)
Alcohol use			
Never	1,825 (16.4)	767 (14.9)	1,058 (17.7)
Ever	8,422 (75.8)	4,030 (78.5)	4,392 (73.4)
Missing	870 (7.83)	336 (6.55)	534 (8.92)
Geographic region			
Europe	5,251 (47.2)	2,323 (45.3)	2,928 (48.9)
North America	4,583 (41.2)	2,254 (43.9)	2,329 (38.9)
South America	1,283 (11.5)	556 (10.8)	727 (12.1)

### SNP selection and validation

Common genetic variants have been identified in Genome Wide Association Studies (GWAS)^21^, ^22^ of 25OHD, only variants that passed a genome wide association threshold (*p* < 5 × 10^−8^) and had been replicated were selected. These genetic proxies or instruments are located in or near four 25OHD related genes: Group‐specific component (*GC*), cytochrome P450 family 2, subfamily R, polypeptide 1 (*CYP2R1*), 7‐dehydrocholesterol reductase (*DHCR7*) and cytochrome P450, family 24, polypeptide 1 (*CYP24A1*). A recent GWAS of 25OHD, a meta‐analysis of 19 studies totalling 42,274 individuals with European ancestry, identified a low frequency variant in *CYP2R1* (rs117913124), independent of the common variant in the same gene.^23^ Effect estimates for all single nucleotide polymorphism (SNP) associations with serum 25OHD concentrations were taken from this GWAS. To assess SNP independence, SNPs on the same chromosome were assessed for linkage disequilibrium (LD) using SNAP (http://archive.broadinstitute.org/mpg/snap/ldsearchpw.php) in Europeans from the 1,000 Genomes (Pilot 1) project. Five SNPs were identified for 25OHD from GWAS (Table 2). rs4588 in *GC*, rs10741657 in *CYP2R1* and rs6013897 in *CYP24A1* have been used in genetic instruments for 25OHD in MR studies before. rs4423214 near *DHCR7* is in perfect LD with rs12785878, identified in the original SUNLIGHT consortium 25OHD GWAS.^22^ Finally, rs116970203 near *CYP2R1* is a low frequency variant identified in the most recent GWAS of 25OHD and has been shown to act independently of rs10741657.^23^

**Table 2:** 25OHD genetic variant details

RSID	Chromosome	Position	Gene	Effect Allele	Other Allele	Vitamin D GWAS				GAME ON	
						EAF	Beta	SE	*p* value	Rsq (minimac3)	EAF
rs4588	4	72618323	*GC*	G	T	0.717	0.2469	0.0070	1.68E‐263	Genotyped	0.722
rs116970203	11	14876718	*PDE3B*	G	A	0.975	0.4323	0.0209	2.29E‐90	0.98	0.978
rs4423214	11	71173254	*DHCR7*	T	C	0.697	0.0998	0.0073	1.39E‐40	0.99	0.718
rs10741657	11	14914878	*CYP2R1*	A	G	0.415	0.0938	0.0065	8.76E‐45	Genotyped	0.383
rs6013897	20	52742479	*CYP24A1*	T	A	0.791	0.0658	0.0080	9.06E‐16	0.77	0.781

Abbeviations: EAF: effect allele frequency; Rsq: R squared; SE: standard error.

In both studies, to correct for within region population structure, region specific principal components were added to the logistic regression models. Effect allele frequencies of the five 25OHD genetic variants were calculated within the three GAME‐ON regions and plotted in pie charts superimposed over a world map.

For each individual, the five 25OHD variants were combined into a genetic risk score. To ease clinical interpretation, this score was converted from a per allele scale to the standardized log 25OHD scale by weighting by the beta coefficients derived from GWAS. Therefore, each unit increase in this variable (hereafter referred to as relative 25OHD) represented a standard deviation (SD) increase in log 25OHD.

### Assessment of potential confounders

One of the principals of MR is that as alleles are assigned randomly during gamete formation and segregation, the genetic instrument is not associated with factors that typically confound the observational association. To test this assumption, associations between the 25OHD instrument and all available potential confounders in GAME‐ON were investigated. Sex, smoking status (never, ever and current) and alcohol use (never and ever) were recorded by questionnaire in both cohorts. GAME‐ON sub‐study and study country were also considered potential confounders.

Associations of the 25OHD instrument with strata of potential confounders was examined in boxplots of range and means ± 1 SD. Tests of associations were made using linear regression with categorical variables as factor variables, the overall *p* values for the model is shown on the boxplots.

### Replication dataset

UK Biobank is a population‐based health research resource consisting of approximately 500,000 people, aged between 38 years and 73 years, who were recruited between the years 2006 and 2010 from across the UK.^24^ Age on 31 December 2010 (coinciding with the approximate end of baseline data collection) was used as the age variable and not age at cancer diagnosis. Prevalent and incident oral and oropharyngeal cancers (ICD10 codes matched to GAME‐ON) were identified from linked cancer registry data. To reduce selection bias in allocating controls, all remaining participants, after removing those participants with other HNC, were used as controls. A full description of the study design, participants and quality control (QC) methods have been described in detail previously.^25^ UK Biobank received ethical approval from the Research Ethics Committee (REC reference for UK Biobank is 11/NW/0382).^26^ In UK Biobank, analyses were restricted to individuals of white British ancestry who self‐report as “White British” and who have very similar ancestral backgrounds according to the principal component analysis, as described by Bycroft.^27^ The full data release contains the cohort of successfully genotyped samples (*n* = 488,377). Any impact of genotyping array was investigated and did not meaningfully affect results (Supporting Information text). Pre‐imputation QC, phasing and imputation are described elsewhere.^27^ Individuals with sex‐mismatch or sex‐chromosome aneuploidy (*n* = 814) and related individuals (*n* = 79,448) were excluded from the analysis (Supporting Information text). After removing related and non‐White British participants, there were 337,108 eligible participants, consisting of 585 cases (oral *n* = 294, oropharyngeal *n* = 291) and 336,523 controls (Supporting Information Table 1).

### Statistical analyses

#### MR analyses

In a method analogous to using a genetic risk score,^28^ relative 25OHD was used in logistic regression models to estimate a causal OR for a SD increase in log 25OHD. The standard error of this estimate was corrected to account for the imprecision of the beta coefficients used to generate the relative 25OHD estimates using a bootstrap technique (Supporting Information text).

##### GAME‐ON

MR analyses were performed within each geographic region of the GAME‐ON consortium accounting for age, sex and the first 15 population specific principal components. As there were likely to be differences between the causal estimates across regions, a random‐effects meta‐analysis was used to combine GAME‐ON causal estimates using the R package ‘*meta*’.^29^ Heterogeneity between study populations was assessed using *I* ^2^.^30^ Meta analyses were repeated excluding geographical regions that were outliers to assess for their influence on the overall estimate.

##### Replication in UK biobank and Meta‐analysis

MR analyses were repeated in UK Biobank. GAME‐ON and UK Biobank estimates were compared and then combined using meta‐analysis. This used a random‐effects model and was conducted using the ‘meta’ package in R.^29^ Heterogeneity between studies was assessed using *I* ^2^.^30^

#### Sensitivity analyses

To assess whether any single SNP was driving a causal estimate, which could be driven by events such as horizontal pleiotropy which invalidate the MR assumptions, a leave‐one out method was applied. This repeats the analysis sequentially removing one SNP from the genetic instrument used to derive relative 25OHD. To further demonstrate this, where a SNP overtly influences an association, each possible genetic instrument from combinations of the five 25OHD SNPs was used to estimate relative 25OHD and thereafter a causal OR. These OR were plotted as histograms highlighting those that include the highly influential SNP to see whether there was an obvious grouping of these estimates.

#### Power

The 25OHD genetic variants have been reported to explain 3–5% of the variance in 25OHD.^21^, ^22^, ^31^ Given this, in the GAME‐ON study for oral (*n* = 2,700 cases, 5984 controls) or oropharyngeal sites (*n* = 2,433 cases, 5984 controls) alone, there was adequate power to detect an OR of 0.67 to 0.74 per SD increase in log 25OHD with power of 0.8 and an alpha of 0.05. For all sites combined (*n* = 5,133 cases, 5984 controls), the study was powered to detect an OR of between 0.74 and 0.79 (http://cnsgenomics.com/shiny/mRnd/).^32^

## Results

Of the five SNPs included in the analyses, rs4588 and rs10741657 were directly genotyped in both the GAME‐ON and UK Biobank studies. All other SNPs were imputed and the imputation quality metric (Minimac3 *r* ^2^ for GAME‐ON; SNPTEST info score for UK Biobank) was >0.98, except rs6013897 in the GAME‐ON study which had an *r* ^2^ of 0.77 (Table 2; Supporting Information Table 2). No two SNPs on the same chromosome had an LD *r* ^2^ >0.01. Allele frequencies were similar across all three regions (Supporting Information Fig. 1).

**Figure 1:**
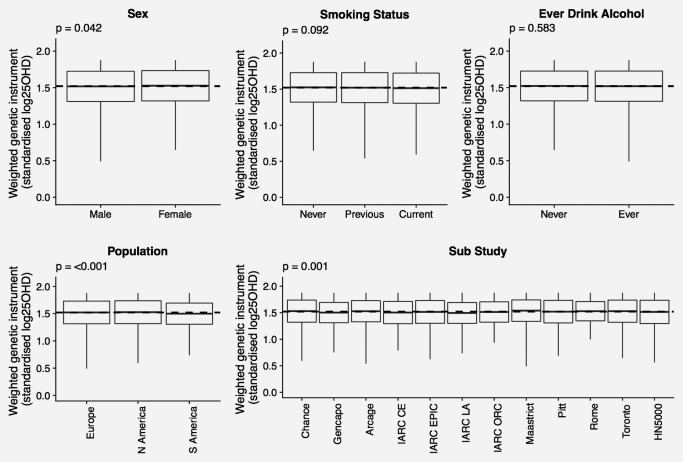
Mean (± SD) and range of 25OHD weighted genetic instrument across strata of potential confounders for GAME‐ON. Overall mean 25OHD weighted genetic instrument for those included in plot indicated by dashed line. Abbreviation: *p*: *p* values for linear model.

In the GAME‐ON study, there was no strong evidence of association of the 25OHD genetic instrument with sex, smoking status or drinking status after taking multiple testing into account. There was evidence for a difference in the 25OHD genetic instrument across regions and sub‐studies (Fig. 1). The region showing deviation from the others was South America and any differences across confounders were no longer seen when the South American region was excluded from analyses (Supporting Information Fig. 2*a*). Within the South American region alone, there was no strong evidence for associations between the 25OHD genetic instrument and confounders (Supporting Information Fig. 2*a*). In UK Biobank, there was no evidence for a difference in relative 25OHD across strata of sex, smoking status or drinking status (Supporting Information Fig. 3).

**Figure 2:**
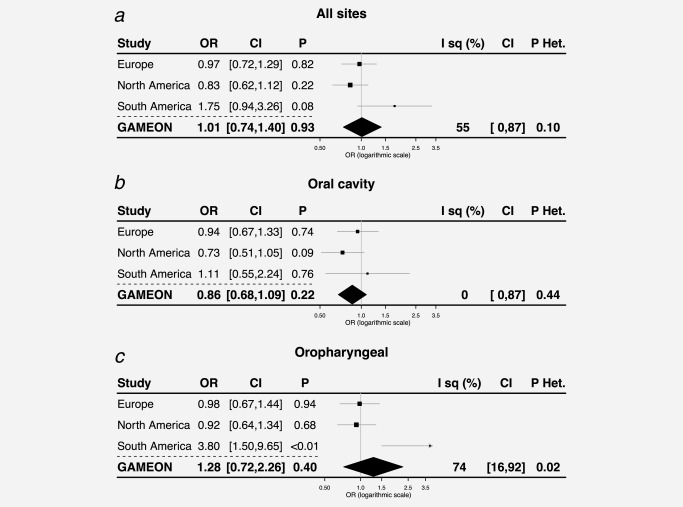
OR (95% CI) for developing cancer for a SD increase in 25OHD for all sites (*a*), oral sites (*b*) and oropharyngeal sites (*c*) in Mendelian randomization analysis. Abbreviations: *p*: *p* values; *p* Het: *p* values for heterogeneity.

In GAME‐ON, for oral and oropharyngeal cancer, there was little evidence for a causal link with relative 25OHD when the three geographic regions were meta‐analysed OR = 0.86 [0.68, 1.09], *p* = 0.22 and OR = 1.28 [0.72; 2.26], *p* = 0.40, respectively. For oral sites, estimates were homogenous (*I* ^2^=0% [0, 87], *p_het_*=0.44) but due to only having three estimates to meta‐analyse (one for each geographic region), power to detect heterogeneity between regions was low. Despite this, there was evidence of heterogeneity between regions for the oropharyngeal sites (*I* ^2^=74% [16, 92], *p_het_*=0.02). This was also reflected in all sites (*I* ^2^=55% [0, 87], *p_het_*=0.10) and was most likely driven by the strong risk increasing association with oropharyngeal cancer identified in the South American region (OR = 3.91 [1.64; 9.29], *p* < 0.01) compared to null associations in both European (OR = 0.98 [0.67; 1.44], *p* = 0.94) and North American regions (OR = 0.92 [0.64; 1.34], *p*0.68) (Fig. 2).

When replicated in UK Biobank, estimates for oral cancer (OR = 0.86 [0.50; 1.51], *p* = 0.61) were very similar with those from the GAME‐ON study. For oropharyngeal sites (OR = 0.85 [0.49; 1.47], *p* = 0.56), point estimates differed between GAME‐ON and UK Biobank although CIs overlapped. When the two studies were meta‐analysed using random‐effects, there was no convincing evidence of a causal association with either cancer or when all sites were analysed (Supporting Information Fig. 4).

### Sensitivity analyses

In leave one out analyses, sequentially omitting each of the five SNPs provided similar causal estimates for any site within the European and North American regions of GAME‐ON. In the South American region of the GAME‐ON study, the risk increasing association between 25OHD and oropharyngeal cancer was most strongly influenced by the rs4588 variant (Supporting Information Fig. 5). In analyses using an instrument with all possible combinations of the 25OHD SNPs, even with the rs4588 variant removed there was still a risk increasing association for oropharyngeal cancer in the South American region (Supporting Information Fig. 6). Removing the South American region from the oropharyngeal analyses reduced heterogeneity between the GAME‐ON studies (OR = 0.95 [0.73, 1.25], *p* = 0.73; *I* ^2^ = 0, *p_het_*=0.82) (Supporting Information Fig. 7).

## Discussion

Our study set out to investigate the causal association between 25OHD and oral and oropharyngeal cancer risk. Given the lack of 25OHD measures in large scale oral and oropharyngeal cancer studies, a two‐sample MR approach,^33^ that allowed genetic variants to be used as largely unconfounded measures of 25OHD, was used. No strong evidence of a causal association was identified with oral or oropharyngeal cancer. Given power calculations predict an OR smaller than 0.74 could be detected, if present any potential true effect is likely to be smaller than this and thus threaten the clinical relevance of the association.

These findings mirror a recently published MR study of colorectal, breast, prostate, ovarian, lung and pancreatic cancer, and neuroblastoma that did not support a causal role of 25OHD in the risk of these cancers.^34^ Similar to the inference from this work on oral and pharyngeal cancer, the authors of the previous study state that although they are unable to rule out clinically relevant effects of small magnitude, their study combined with previous literature, ‘provide evidence that population‐wide screening for vitamin D deficiency and subsequent widespread vitamin D supplementation should not currently be recommended as a strategy for primary cancer prevention’. Recent RCTs examining the effect of vitamin D on cancer incidence do not show strong evidence of a protective effect, although these studies are limited to older females.^35^, ^36^ Contrary to this, Ong *et al*.^18^ did demonstrate a protective role for 25OHD in ovarian cancer in a well powered MR study. Despite the body of evidence that does not support a protective effect of 25OHD on cancer incidence, there is evidence to support the hypothesis that vitamin D has an effect on cancer progression.^8^, ^11^

The genetic instrument used in our study specifically proxies average total (free and bound) circulating 25OHD and does not necessarily predict the concentrations of free 25OHD available at the tissues or concentrations of free or bound 1,25OH_2_D in circulation or at the tissue level. Potential anti‐cancer effects of vitamin D are via free 1,25OH_2_D interacting with the vitamin D receptor within tissues resulting in reduced angiogenesis, metastasis, cell invasion, inflammation, and proliferation as well as upregulation of apoptosis.^8^ Levels of 1,25OH_2_D are assumed to be correlated with circulating 25OHD, but this is not necessarily the case. Although less likely than a null association between 25OHD and oral and oropharyngeal cancer risk, the instrument being invalid for 25OHD could explain the disparity between the results of our study and previous findings showing a protective effect of 25OHD. However, as the genetic variants used here as instruments for 25OHD have robust associations with 25OHD and are in genes known to effect this metabolite they are likely to be valid for the purposes of MR.

To be valid, the genetic instrument must proxy circulating 25OHD without affecting cancer risk through other casual pathways (violation of this assumption is referred to as horizontal pleiotropy). The SNPs used in this instrument are in genes with known effects on vitamin D pathways and have been consistently associated with circulating levels of 25OHD in GWA^21^, ^22^ and MR studies,^37^ reducing the chance of horizontal pleiotropy. Sensitivity analyses such as MR Egger^38^ and the weighted median method^39^ can detect or correct for the presence of directional pleiotropy but have low power with few SNPs comprising the genetic instrument, as in this case. Here, causal estimates in the European and North American populations are largely consistent across all five SNPs, providing some evidence against the presence of strong pleiotropy that could bias findings.

In the South American population, a risk increasing association was identified between 25OHD and oropharyngeal cancer. Use of individual level data in our study allowed scrutiny of the genetic instrument‐confounder independence assumption and showed that the 25OHD instrument generally held up to this assumption; however, there was some evidence the genetic instrument was associated with region and sub‐study, driven by the South American region. Given the above points, it is unlikely then that pleiotropy or confounding can explain the large risk increasing association seen for oropharyngeal cancer in the South American region. However, as this risk increasing association is inconsistent with observational effect directions and MR estimates from other geographic regions, it is unlikely to be truly causal: it most likely represents structure within this population that is by chance associated with oropharyngeal cancer risk. To assess the impact of this potentially biased estimate on the overall causal OR, the analyses were repeated with the South American individuals removed, resulting in more consistent estimates.

## Conclusions

Our study does not support the observational association between 25OHD and oral cancer risk and is consistent with evidence that a causal, clinically relevant protective effect of 25OHD on oropharyngeal cancer risk is unlikely. The effect of 25OHD on oral and oropharyngeal cancer progression was not assessed here and requires further investigation.

The genetic instrument for 25OHD is a good proxy for circulating total 25OHD, the same metabolite that is used in supplementation and is measured in the observational studies. Despite being robustly associated with 25OHD, the genetic variants used here only explain a relatively small amount of variance in 25OHD. This means that causal effects of small magnitude cannot be ruled out. Furthermore, it cannot be determined whether 25OHD has an effect on cancer risk in individuals below a certain 25OHD concentration threshold. Where inference is likely to be useful clinically is in relation to supplementation. Supplementation increases total circulating 25OHD and is presumed to subsequently increase free 1,25OH_2_D availability at the tissues. The SD 25OHD increase in our study will have a relative effect on total 25OHD across the whole life course. If this long‐term difference in relative 25OHD does not produce a detectable effect in our study, any 25OHD causal effect is unlikely to be of a magnitude that would warrant long‐term supplementation in the general population from a particular age.

## Supporting information

Supporting Figures

Supplementary Table 1: UK Biobank participant summaries

Supplementary table 2: 25‐Hydroxyvitamin D genetic variant details
