# Supplementary material for: Assessing the causal association between 25‐hydroxyvitamin D and the risk of oral and oropharyngeal cancer using Mendelian randomization
Source: Int J Cancer. 2018 Jul 30;143(5):1029–36. doi: 10.1002/ijc.31377 (PMC6099266; doi:10.1002/ijc.31377)
Supplement: Supplementary file 2 — Supplementary Table 1: UK Biobank participant summaries [file IJC-143-1029-s002.docx]

| **Supplementary table 1: UK Biobank participant summaries** | | | |
| --- | --- | --- | --- |
|  | All n (%) | Case n (%) | Control n (%) |
|  | N=337108 | N=585 | N=336523 |
| Site |  |  |  |
| Oral |  | 294 (50.3) |  |
| Oropharyngeal |  | 291 (49.7) |  |
| Age |  |  |  |
| =<50 | 65349 (19.4%) | 51 (8.72%) | 65298 (19.4%) |
| 50-<60 | 94102 (27.9%) | 180 (30.8%) | 93922 (27.9%) |
| 60-<70 | 153829 (45.6%) | 298 (50.9%) | 153531 (45.6%) |
| >=70 | 23828 (7.07%) | 56 (9.57%) | 23772 (7.06%) |
| Missing |  |  |  |
| Sex |  |  |  |
| Male | 155747 (46.2%) | 387 (66.2%) | 155360 (46.2%) |
| Female | 181361 (53.8%) | 198 (33.8%) | 181163 (53.8%) |
| Smoking status |  |  |  |
| Never | 184081 (54.6%) | 208 (35.6%) | 183873 (54.6%) |
| Previous | 118478 (35.1%) | 267 (45.6%) | 118211 (35.1%) |
| Current | 33364 (9.90%) | 107 (18.3%) | 33257 (9.88%) |
| Missing | 1185 (0.35%) | 3 (0.51%) | 1182 (0.35%) |
| Alcohol use |  |  |  |
| Never | 10406 (3.09%) | 10 (1.71%) | 10396 (3.09%) |
| Ever | 326467 (96.8%) | 575 (98.3%) | 325892 (96.8%) |
| Missing | 235 (0.07%) | 0 (0.00%) | 235 (0.07%) |
|  |  |  |  |
|  |  |  |  |

| **Supplementary table 2: 25-Hydroxyvitamin D genetic variant details** | | | | | |  |  |  |  |  |  |  |
| --- | --- | --- | --- | --- | --- | --- | --- | --- | --- | --- | --- | --- |
|  |  |  |  |  |  | Vitamin D GWAS | | | |  | UK Biobank | |
| RSID | Chromosome | Position | Gene | Effect Allele | Other Allele | EAF | Beta | SE | P value |  | Info (snptest) | EAF |
| rs4588 | 4 | 72618323 | *GC* | G | T | 0.717 | 0.2469 | 0.0070 | 1.68E-263 |  | Genotyped | 0.712 |
| rs116970203 | 11 | 14876718 | *PDE3B* | G | A | 0.975 | 0.4323 | 0.0209 | 2.29E-90 |  | 0.99 | 0.973 |
| rs4423214 | 11 | 71173254 | *DHCR7* | T | C | 0.697 | 0.0998 | 0.0073 | 1.39E-40 |  | 1.00 | 0.761 |
| rs10741657 | 11 | 14914878 | *CYP2R1* | A | G | 0.415 | 0.0938 | 0.0065 | 8.76E-45 |  | Genotyped | 0.398 |
| rs6013897 | 20 | 52742479 | *CYP24A1* | T | A | 0.791 | 0.0658 | 0.0080 | 9.06E-16 |  | 1.00 | 0.805 |
| EAF = effect allele frequency, Rsq = R squared, SE = standard error | | | | | | | | | | | | |
