# Supplementary material for: Assessing the causal association between 25‐hydroxyvitamin D and the risk of oral and oropharyngeal cancer using Mendelian randomization
Source: Int J Cancer. 2018 Jul 30;143(5):1029–36. doi: 10.1002/ijc.31377 (PMC6099266; doi:10.1002/ijc.31377)
Supplement: Supplementary file 3 — Supplementary table 2: 25‐Hydroxyvitamin D genetic variant details [file IJC-143-1029-s003.docx]

**Supplementary Text**

**Genotyping and imputation**

*GAMEON*

Genomic DNA isolated from blood or buccal cells was genotyped using the Illumina OncoArray custom designed by the OncoArray Consortium for cancer studies^1^. All samples were genotyped as part of the oral and pharynx cancer OncoArray, apart from 2,476 shared controls that were genotyped as part of the Lung cancer OncoArray. After single nucleotide polymorphism (SNP) filtering by call rate, 513,311 probes remained for analysis. Within each geographical area, principal component were generated using EIGENSTRAT^2^. Imputation of unknown genetic variation was performed on the Michigan Imputation Server^3^ using the first release of the Haplotype Reference consortium panel.

*UKBIOBANK*

Pre-imputation QC, phasing and imputation

Prior to phasing, multiallelic single nucleotide polymorphisms (SNPs) or those with minor allele frequency ≤1% were removed. Phasing of genotype data was performed using a modified version of the SHAPEIT2 algorithm.^4^ Genotype imputation to a reference set combining the UK10K haplotype and Haplotype Reference Consortium reference panels^5^ was performed using IMPUTE2 algorithms.^6^

Post imputation QC.

Individuals with sex-mismatch (derived by comparing genetic sex and reported sex) or individuals with sex-chromosome aneuploidy were excluded from the analysis (n=814). Estimated kinship coefficients using the KING toolset^7^ identified 107,162 pairs of related individuals.^8^ An in-house algorithm was then applied to this list and preferentially removed the individuals related to the greatest number of other individuals until no related pairs remain. These individuals were excluded (n=79,448). Additionally 2 individuals were removed due to them relating to a very large number (>200) of individuals.^9^

Principal components were generated by UK BIOBANK using flashPCA.

**Two-sample Mendelian randomization analysis (individual level data) with bootstrap correction of standard errors.**

To account for the imprecision of the beta coefficients used to generate the relative 25OHD estimates, a bootstrap technique was applied with 500 iterations using the ‘boot’ package in R.^10^ 500 imputed relative 25OHD values were generated from a sampling distribution based on the mean and SD from the 25OHD GWAS study. This set of newly imputed relative 25OHD values were used in logistic regression models with cancer case status as the outcome to generate a distribution of causal estimates. The standard deviation of this distribution was added to the standard error of the original casual OR to give a corrected standard error.

- 1. STAGE 1: Create estimate of vitamin D in each participant using Beta from GWAS and dosage of variant
  $\alpha_{1\ldots j}=Beta from GWAS for {variant}_{1\ldots j}$
  $Z_{i}= Dosage for {variant}_{1\ldots j} in individual$
  $\hat{X}_{i}=estimate of standardised change in\log vitamin D using {variant}_{1\ldots j}$
  $\hat{X}_{i}= \alpha_{1}Z_{1i}+\ldots+\alpha_{j}Z_{ji}$
  2. STAGE 2: Estimate causal odds ratio of vitamin D on OC/OPC risk (for each SNP) as the coefficient of the below logistic regression

$p_{i}=probability of OC/OPC$ in individual

- 1. CORRECTED STANDARD ERRORS: The SE from stage 2 will not consider the error in *α_1…j_*, a bootstrap method will be used to generate corrected SE for the second stage regression.

$$B=number of bootstraps (500)$$

$for b=1 to B; Generate \hat{X}_{i}^{*}$

$$\hat{X}_{i}^{*b}= \alpha_{j}^{*b}Z_{1i}+\ldots+\alpha_{j}^{*b}Z_{ji} , \alpha_{j}^{*b}\sim(\alpha_{j}, s{e\left( \alpha_{j} \right)}^{2})$$

$Generate distribution of bootstraped causal OR \left( \psi^{*} \right)$

$$logit\left( p_{i} \right)= \beta_{0}+\psi^{*}\hat{X}_{i}^{*}$$

$Corrected se\left( \psi\right)\approx se\left( \psi\right)+se(\psi^{*})$

**Impact of UK BIOBANK genotyping array**

A nested focus study (UKBiLEVE),^11^ investigating lung disease, preferentially selected and genotyped heavy and non-smokers using a different array to the remainder of the participants. 49,979 individuals were genotyped using the UK BiLEVE array and 438,398 using the UK Biobank axiom array. Removing UKBiLEVE participants did not alter the MR results markedly and the interpretation without them would be identical **(Supplementary Figure 8)**.

**References**

1. Consortium Launches Genotyping Effort. *Cancer Discov.* **3,** (2013).

2. Price, A. L. *et al.* Principal components analysis corrects for stratification in genome-wide association studies. *Nat. Genet.* **38,** 904–909 (2006).

3. Das, S. *et al.* Next-generation genotype imputation service and methods. *Nat. Genet.* **48,** 1284–1287 (2016).

4. O’Connell, J. *et al.* Haplotype estimation for biobank-scale data sets. *Nat. Genet.* **48,** 817–820 (2016).

5. Huang, J. *et al.* Improved imputation of low-frequency and rare variants using the UK10K haplotype reference panel. *Nat. Commun.* **6,** 8111 (2015).

6. Howie, B., Marchini, J. & Stephens, M. Genotype Imputation with Thousands of Genomes. *G3 Genes, Genomes, Genet.* **1,** 457–470 (2011).

7. Manichaikul, A. *et al.* Robust relationship inference in genome-wide association studies. *Bioinformatics* **26,** 2867–2873 (2010).

8. Bycroft, C. *et al.* Genome-wide genetic data on ~500,000 UK Biobank participants. *bioRxiv* 166298 (2017). doi:10.1101/166298

9. Mitchell, R., Hemani, G., Dudding, T. & Paternoster, L. *UK Biobank Genetic Data: MRC-IEU Quality Control, Version 1*. (University of Bristol, 2017). doi:10.5523/bris.3074krb6t2frj29yh2b03x3wxj

10. Canty, A. & Ripley, B. boot: Bootstrap R (S-Plus) Functions. R package version 1.3-20. 2017.

11. Wain, L. V *et al.* Novel insights into the genetics of smoking behaviour, lung function, and chronic obstructive pulmonary disease (UK BiLEVE): a genetic association study in UK Biobank. *Lancet Respir. Med.* **3,** 769–781 (2015).
